# COVID-19 related anxiety and its associated factors: a cross-sectional study on older adults in Bangladesh

**DOI:** 10.1186/s12888-022-04403-2

**Published:** 2022-11-28

**Authors:** Sabuj Kanti Mistry, ARM Mehrab Ali, Uday Narayan Yadav, Sukanta Das, Nahida Akter, Md. Nazmul Huda, Setho Hadisuyatmana, Sajedur Rahman, David Lim, Mohammad Mahmudur Rahman

**Affiliations:** 1 ARCED Foundation, 13/1 Pallabi, Mirpur-12, Dhaka, 1216 Bangladesh; 2 Centre for Primary Health Care and Equity, University of New South Wales, Sydney, NSW 2052 Australia; 3 Department of Public Health, Daffodil International University, Dhaka, 1207 Bangladesh; 4 National Centre for Epidemiology and Population Health, Research School of Population Health, The Australian National University, Canberra, ACT Australia; 5 Department of Statistics, Begum Rokeya University, Rangpur, Bangladesh; 6 Maternal and Child Health Division, International Centre for Diarrhoeal Disease Research, (ICDDR,B), Dhaka, Bangladesh; 7 School of Medicine, Translational Health Research Institute, Western Sydney University, Campbeltown, NSW Australia; 8 The Faculty of Nursing, Universitas Airlangga, Surabaya, 60115 Indonesia; 9 Save the Children in Bangladesh, Dhaka, 1212 Bangladesh; 10 School of Health Sciences, Western Sydney University, Campbeltown, NSW Australia; 11 Department of Clinical Psychology, University of Dhaka, Dhaka, Bangladesh

**Keywords:** Coronavirus anxiety, COVID-19, Older adults, Bangladesh

## Abstract

**Background:**

The COVID-19 pandemic has resulted in serious mental health conditions, particularly among older adults. This research explored the prevalence of COVID-19-related anxiety and its associated factors among older adults residing in Bangladesh.

**Methods:**

This cross-sectional study was conducted among 1,045 older Bangladeshi adults aged ≥ 60 years through telephone interviews in September 2021. A semi-structured interview schedule was used to collect data on participants’ characteristics and COVID-19-related anxiety. The anxiety level was measured using the Bengali version of the five-point Coronavirus Anxiety Scale (CAS). A linear regression model explored the factors associated with COVID-19-related anxiety.

**Results:**

Overall, the prevalence of COVID-19-related anxiety was 23.2%. The regression analysis revealed that the average COVID-19-related anxiety score was significantly higher among females (β: 0.43, 95% CI: 0.05 to 0.81), and among those who faced difficulty getting medicine (β: 0.57, 95% CI: 0.16 to 0.97), felt isolated (β: 0.60, 95% CI: 0.24 to 0.95), and felt requiring additional care during the pandemic (β: 0.53, 95% CI: 0.16 to 0.91). Alternatively, the average COVID-19-related anxiety score was significantly lower among those who were widowed (β: -0.46, 95% CI: -0.87 to -0.04) and living distant from the health centre (β: -0.48, 95% CI: -0.79 to -0.17).

**Conclusion:**

The findings of the present study suggest providing immediate psychosocial support package to the older adults, particularly females and those who are vulnerable to receive health and social care support during the COVID-19 pandemic in Bangladesh.

**Supplementary Information:**

The online version contains supplementary material available at 10.1186/s12888-022-04403-2.

## Background

The COVID-19 pandemic is a major global health crisis of the century [1]. Since the declaration of the pandemic in March 2020, there are more than 628 million confirmed cases and more than 65 million deaths internationally as of 4 November 2022 [2]. Like many countries of the global South, Bangladesh is heavily affected by the pandemic [3, 4][5,6]. As of 4 November 2022, there were more than two million confirmed COVID-19 cases and 29,425 deaths in Bangladesh [2]. In particular, older people are at increased risk of COVID-19 related adverse events and mortality in Bangladesh [7] due to their concomitant non-communicable diseases, such as diabetes mellitus, hypertension, obesity, and cardiovascular problems [8]. While there is limited age-specific COVID-19 data available in Bangladesh, evidence indicates that nearly 45% of the total COVID-19 deaths occurred among older adults [7].

The prolonged pandemic not only hampered the physical conditions of the population, but existing evidence also indicates that it has resulted in severe psychological consequences, including stress, fear, and anxiety among the population worldwide [9, 10]. Several studies conducted in overseas documented that the prevalence of anxiety was higher among older people during the COVID-19 pandemic compared to that of the pre-pandemic [11, 12, 13, 14]. Globally, the prevalence of COVID-19-related anxiety was 24% among the older population [15], with the highest proportions reported in low- and middle-income countries (LMICs) [16]. This can contribute to an additional burden to what is already existing among older people, accounting for 6.6% of the total disability-adjusted life years in people aged 60 years and above [17].

COVID-19-related fear and anxiety was precipitated by public health measures implemented to contain the spread of the SARS-CoV-2 virus. For instance, earlier studies reported that COVID-19-related lockdown is a contributing factor of the increased mental health problems in the older population [9, 12][18,19]. These studies further documented that limited direct contact with friends and family members and inadequate support during the pandemic exacerbated their mental health conditions. The level of fear and anxiety were also higher in those who had poor health [20], lived with chronic non-communicable diseases and had limited access to medications [9][19]. Additionally, COVID-19-related anxiety was more common in females living alone than males [9, 13, 14,19,21].

While previous studies documented the level of fear among older adults in Bangladesh [4, 22], there are limited studies conducted to explore COVID-19-related anxiety among older adults. Most studies examined students’ anxiety and depression using online questionnaires [23, 24, 25]. For example, Islam and colleagues (2020) found that 18% students suffered from anxiety, while older students tended to have more depressive symptoms than others [24]. A more recent study examined the impact of COVID-19 on older people and reported 34% of anxiety symptoms among adult people in Bangladesh [26]. However, to the best of our knowledge, no study comprehensively examined COVID-19-related anxiety and its associated factors among the older people in Bangladesh, using an established scale such as Coronavirus Anxiety Scale (CAS). Therefore, the current study aims to explore the prevalence of COVID-19-related anxiety and its associated factors among older adults residing in Bangladesh.

The findings of our study are critical to inform existing mental health interventions during the COVID-19 pandemic and beyond. Mental health is a severely neglected issue in Bangladesh, specifically among older people. The pandemic disrupted the already existing inadequate mental health services [27], especially among older people [28], increasing their vulnerability to anxiety and depression. Evidence indicates that the COVID-19 pandemic and its impacts on Bangladeshi older people have not ended yet due to several factors, including but not limited to, inadequate and untimely measures for controlling coronavirus infection [7], low vaccination coverage among older people [29], irregularities, and limited preparedness of the health sector [30]. In such a situation, severe psychological problems have been reported, triggering thoughts of suicide among individuals, including older people [1, 31]. The unresolved fears due to the history of a family being infected with COVID-19, potential infection, decreased income, food insecurity and inability to maintain COVID-19 preventive measures may further trigger older people’s adverse mental health conditions during this pandemic [3, 4, 11]. Therefore, a better understanding of COVID-19-related anxiety and its related factors is crucial to inform interventions related to Bangladeshi older people’s psychological wellbeing. Thus, the present study was designed to fill the knowledge gap by thoroughly investigating COVID-19-related anxiety and its associated factors among the older people in Bangladesh.

## Subjects and methods

### Study design and participants

This cross-sectional study was conducted during September and October 2021 among older adults aged 60 years and above residing in Bangladesh. We utilized our pre-established registry as a sampling frame used in our previous studies [32], which included households from all eight administrative divisions of Bangladesh. Based on the population distribution of older adults by geography in Bangladesh, we adopted a probability proportionate to size (of the eight-division) approach to select older adults in each division [33]. Considering 50% prevalence with a 5% margin of error, at the 95% level of confidence, 90% power of the test, and 95% response rate, a sample size of 1096 was calculated. Based on the probability proportionate to size of the eight-divisions we calculated a sample size required for each of the division and was randomly selected from the list of the participants of the registry. However, 1045 of the approached eligible participants responded to the study with an overall response rate of approximately 95%. The inclusion criterion was the minimum age of 60 years, and the exclusion criteria were adverse mental conditions (clinically diagnosed schizophrenia, bipolar mood disorder, dementia/cognitive impairment), a hearing disability, or an inability to communicate.

We recruited research assistants having previous experience in health data collection on electronic platforms. The research assistants were trained in the Zoom platform for three days and data collection was accomplished in SurveyCTO mobile platform (https://www.surveycto.com/). We used a pre-tested semi-structured interview schedule for the data collection. The developed interview schedule was piloted with ten older adults to refine the language in the final version. However, no corrections/suggestions were received from the participants. The data collection was accomplished using this final version of the interview schedule through a telephone interview.

### Measures

#### Outcome measure

The outcome variable for the study was the level of COVID-19-related anxiety, measured using the five-point CAS [34]. The scale was also validated to the Bengali language [35]. Participants were asked about the level of COVID-19-related anxiety they experienced in the last two weeks preceding the survey on the five CAS items and their agreement/disagreement with these items were assessed using a five-point Likert Scale. Hence, the cumulative score ranged from 0 to 20, where the higher the scores, the greater the anxiety of COVID-19. We further classified the participants as having COVID-19-related anxiety (if they reported having anxiety in any one of the CAS items) or not having COVID-19-related anxiety (if they reported they had no anxiety in every CAS items). We found the reliability of the scale among the participant acceptable (Cronbach’s α = 0.84).

#### Explanatory variables

The explanatory variables used in the study were sex (male/female), age in years (60–69, and ≥ 70), receiving formal education (no/yes), marital status (married/widowed), family income in Bangladeshi Taka (BDT) (< 5,000, 5,000–10,000, > 10,000), family size (≤ 4 or > 4), current occupation (employed/unemployed), residence (urban/rural), living arrangements (living alone or with family), having memory or concentration problems (no problem/low memory or concentration), walking distance to the nearest health center (< 30 min/ ≥ 30 min), presence of non-communicable chronic conditions (no/yes), overwhelmed by COVID-19 (hardly, sometimes/often), concerned about COVID-19 (hardly, sometimes/often), difficulty in earning during COVID-19 (no/yes), frequency of communication with friends and family during COVID-19 (less than previous/same as previous), perceived isolation from others during OVID-19 (hardly, sometimes/often), perceived that family members are non-responsive during COVID-19 (no/yes), and perceived that they required additional care during COVID-19 (no/yes).

According to the most recent Household Income and Expenditure Survey (HIES), the average family size in Bangladesh was 4.1. Therefore, we categorized the family size as ≤ 4 or > 4 [36]. Pre-existing medical conditions were self-reported in this study (e.g., hypertension, arthritis, stroke, heart diseases, diabetes, chronic respiratory diseases, hypercholesterolemia, chronic kidney disease, and cancer). Thereafter, we created a new variable, “presence of non-communicable chronic conditions,” which was categorized as “No” if they did not have any of these diseases and “Yes” if they had at least one of these diseases.

### Statistical analysis

The distribution of the variables was assessed through descriptive statistics. A linear regression model was performed to explore the factors associated with anxiety among the participants. We performed an initial model with all potential covariates. Thereafter, using the backward elimination criteria with the Akaike information criterion (AIC), variables for the final regression model were selected and executed. In this case, the adjusted beta coefficient, p-value, and 95% confidence interval (95% CI) for the final model are reported in the main table, and the model multicollinearity diagnostics results are presented in a supplementary table. All analyses were performed using the statistical software package Stata (Version 17.0).

## Results

### Characteristics of the participants

A total of 1045 adults aged 60 and over participated in this study from the eight administrative divisions of Bangladesh. Table 1 represents the participants’ socio-demographic characteristics and perceived opinions on COVID-19-related information. The majority of the respondents were aged 60–69 years (75.6%), male (59.3%), married (76.5%), unemployed (61.1%), living in rural areas (82.6%), living with family members (94.9%), and had a large family with more than four members (66.8%). Nearly half of the participants (48.3%) had formal schooling, had a monthly family income of 5,000–10,000 BDT (44.9%), and resided more than 30 min of walking distance from the nearest health center (44.4%). More than half of the respondents (57.2%) were suffering from any non-communicable chronic condition, and they were sometimes or often feeling concerned (66.7%) and overwhelmed (67.6%) by COVID-19. Approximately a quarter of the participants (24.7%) informed of experiencing difficulty getting medicine during COVID-19. Around two-fifths of the participants (37.2%) reported that the frequency of communication with friends and family during the pandemic was less than the previous. Moreover, many participants also reported feeling isolated (31.1%), feeling family members were non-responsive (29.4%) and requiring additional care (26.3%) during the COVID-19 pandemic.

**Table 1:** Characteristics of the participants and bivariate analysis (N = 1045)

Characteristics	n	%		Anxiety				
				No		Yes	*P*	
Administrative division								
Barishal	146		14.0		69.9	30.1		0.025
Chattogram	98		9.4		81.6	18.4		
Dhaka	172		16.5		79.1	20.9		
Mymensingh	69		6.6		81.2	18.8		
Khulna	198		19.0		70.2	29.8		
Rajshahi	145		13.9		84.1	15.9		
Rangpur	161		15.4		78.3	21.7		
Sylhet	56		5.4		75.0	25.0		
Age (year)								
60—69	790		75.6		77.7	22.3		0.236
> = 70	255		24.4		74.1	25.9		
Sex								
Male	620		59.3		79.0	21.0		0.043
Female	425		40.7		73.7	26.4		
Marital status								
Married	799		76.5		77.4	22.7		0.486
Widowed	246		23.5		75.2	24.8		
Formal schooling								
No formal schooling	540		51.7		73.7	26.3		0.013
Having formal schooling	505		48.3		80.2	19.8		
Family size								
≤ 4	347		33.2		80.1	19.9		0.077
> 4	698		66.8		75.2	24.8		
Family monthly income (BDT)								
< 5000	121		11.6		71.9	28.1		0.104
5000–10,000	469		44.9		75.3	24.7		
> 10,000	455		43.5		79.8	20.2		
Residence								
Urban	182		17.4		79.1	20.9		0.423
Rural	863		82.6		76.4	23.6		
Current occupation								
Employed	407		39.0		80.1	19.9		0.046
Unemployed	638		61.1		74.8	25.2		
Living arrangement								
Living with family	992		94.9		77.0	23.0		0.564
Living alone	53		5.1		73.6	26.4		
Walking distance to the nearest health centre								
< 30 min	581		55.6		74.2	25.8		0.023
≥ 30 min	464		44.4		80.2	19.8		
Problem in memory or concentration								
No problem	676		64.7		78.3	21.8		0.143
Low memory or concentration	369		35.3		74.3	25.8		
Suffering from non-communicable chronic conditions								
No	447		42.8		81.4	18.6		0.002
Yes	598		57.2		73.4	26.6		
Feeling concerned about COVID-19								
Hardly	348		33.3		84.8	15.2		0.000
Sometimes/often	697		66.7		72.9	27.1		
Feeling overwhelmed by COVID-19								
Hardly	334		32.1		82.0	18.0		0.006
Sometimes/often	706		67.9		74.4	25.6		
Difficulty in getting medicine during COVID-19								
No	764		74.8		80.6	19.4		0.000
Yes	258		25.2		65.9	34.1		
Frequency of communication during COVID-19								
Same as previous	656		62.8		77.0	23.0		0.890
Less than previous	389		37.2		76.6	23.4		
Feeling isolated from others								
Hardly	718		68.7		81.1	18.9		0.000
Sometimes/often	327		31.3		67.6	32.4		
Feeling that family members are non-responsive								
No	738		70.6		77.8	22.2		0.266
Yes	307		29.4		74.6	25.4		
Feeling that they required additional care during the pandemic								
No	770		73.7		80.4	19.6		0.000
Yes	275		26.3		66.9	33.1		

1 BDT ~ 0–0.010 USD

### Prevalence of anxiety

Overall, the prevalence of anxiety was 23.2% among the participants. The prevalence of anxiety was significantly higher among females (26.4%), unemployed (25.2%), those resided near to the health centre (25.8%), those suffering from chronic conditions (26.6%), those who were concerned about COVID-19 (27.1%), those facing difficulty getting medicine during the pandemic (34.1%), those who felt isolated (32.4%) and perceived needing additional care (33.1%) during the pandemic. Details are presented in Table 1. Meanwhile, the percentage of participants with responses to individual items of the CAS is presented in Annex 1.

### Factors associated with anxiety

The socio-demographic characteristics of the participants and COVID-19-related information (Table 1), which were deemed to be associated with COVID-19-related anxiety, were included in the initial linear regression model. After that, a final model was performed, including all the variables retained from the initial model based on the lowest AIC. The result from the final regression model is presented in Table 2. All independent variables were checked for multicollinearity, and no significant multicollinearity was observed for any variable (Annex 2).

**Table 2:** Factors associated with anxiety among the participants (*N* = 1045)

Characteristics	Unadjusted			Adjusted		
	β	*P*	95%CI	β	*P*	95%CI
Sex						
Male	ref			ref		
Female	0.36	**0.036**	0.02, 0.69	0.43	**0.026**	0.05, 0.81
Marital status						
Married	ref			ref		
Widowed	-0.08	0.668	-0.44, 0.28	-0.46	**0.032**	-0.87, -0.04
Formal schooling						
No formal schooling	ref			ref		
Having formal schooling	-0.33	0.043	-0.65, -0.01	-0.31	0.054	-0.62, 0.01
Family size						
≤ 4	ref			ref		
> 4	0.27	0.095	-0.05, 0.60	0.26	0.113	-0.06, 0.57
Walking distance to the nearest health centre						
< 30 min	ref			ref		
≥ 30 min	-0.48	**0.003**	-0.79, -0.17	-0.48	**0.002**	-0.79, -0.17
Feeling concerned about COVID-19						
Hardly	ref			ref		
Sometimes/often	0.49	**0.003**	0.16, 0.81	0.26	0.108	-0.06, 0.58
Difficulty in getting medicine during COVID-19						
No	ref			ref		
Yes	0.80	** < 0.001**	0.38, 1.22	0.57	**0.006**	0.16, 0.97
Feeling isolated from others						
Hardly	ref			ref		
Sometimes/often	0.83	** < 0.001**	0.45, 1.20	0.60	**0.001**	0.24, 0.95
Feeling that they required additional care during the pandemic						
No	ref			ref		
Yes	0.69	**0.001**	0.30, 1.09	0.53	**0.005**	0.16, 0.91

The adjusted regression model revealed that the average COVID-19-related anxiety score was significantly higher among females than males (β: 0.43, 95% CI: 0.05 to 0.81). Similarly, the anxiety score was significantly higher among participants who faced difficulty in getting medicine (β: 0.57, 95% CI: 0.16 to 0.97), felt isolated from others (β: 0.60, 95% CI: 0.24 to 0.95) and felt requiring additional care during the COVID-19 pandemic (β: 0.53, 95% CI: 0.16 to 0.91). On the other hand, the COVID-19-related anxiety score was significantly lower among those who were widowed (β: -0.46, 95% CI: -0.87 to -0.04) and for whom the nearest health centre was at more than 30-min walking distance (β: -0.48, 95% CI: -0.79 to -0.17).

## Discussion

The current study aimed to explore the level of COVID-19-related anxiety among the older adults in Bangladesh during this COVID-19 pandemic using the translated CAS instrument. The study found that around one-quarter of the Bangladeshi older adults (23.2%) had COVID-19-related anxiety. We did not find any study that reports the COVID-19-related anxiety level among the older population during the COVID-19 pandemic in Bangladesh using the CAS. However, a recent study among older people in India using the Geriatric Anxiety Scale [37,38] reported an anxiety prevalence of 8.7%, which is lower to what we have found in our study. Existing Bangladeshi studies on University students [23, 24], young people [39], health workers [40] and the general population [41, 42] reported anxiety prevalence during the COIVD-19 pandemic, ranging from 40 to 70%. This prevalence is higher than what we found in the current study. Several factors, such as, different measurement tools, sampling variations, heterogeneity of age range, various risk factors, pandemic and pre-pandemic situations, and socio-cultural variations may explain the differences in older people’s anxiety levels in these studies.

The present study revealed that many participants reported becoming anxious about COVID-19 when they read or listened to news about the coronavirus. This is probably due to a higher level of misconception related to COVID-19 among older people fueled by misinformation and rumors speeded in Bangladesh. A recent study also documented a high level of COVID-19-related misconceptions among the older adults in Bangladesh. Evidence suggests that following COVID-19-related news and fear of infection were significantly associated with anxiety and stress among Bangladeshi people [43]. Considering this, the National Institute of Mental Health of Bangladesh has recommended avoiding COVID-19 news or scrolling the news several times a day, authenticating sources of information, and limited use of social media to preserve mental health during the COVID-19 pandemic [41]. It is also important to mention that, because of the limited digital literacy among the older population, it is often difficult for them to receive COVID-19-related information on digital platforms [44]. Moreover, most older adults have limited access to internet services and smartphones, and as such, only a small fraction of older adults can benefit from such service provisions [45]. They often find it challenging to navigate through the digital world and understand the news’s proper meaning, which might cause increased anxiety. Our study’s findings suggest disseminating anxiety reduction messages via community leaders and health staff during the pandemic.

We found that the COVID-19-related anxiety score was significantly higher among the female participants. This finding is similar to a previous study conducted in Bangladesh, which showed that female older adults were more likely to have anxiety disorder due to COVID-19 [42]. In Bangladesh, most women are still dependent on their husbands for their livelihoods due to their relatively limited engagement in the job sectors [46]. This dependency on male partners increases their risk of financial insecurity, resulting in an increased level of anxiety. Among older females, anxiety can also stem from their experience of health-related problems. Compared to younger individuals, female senior citizens in Bangladesh suffer from health-related problems [47], which may increase their anxiety during the COVID-19 pandemic. However, contradicting the findings reported elsewhere [48, 49], we found that unmarried participants had less anxiety than those currently married. This is possibly because single participants had less chance to discuss the risks associated with COVID-19 with their family members, thus making them less conscious about the adverse effects of COVID-19, including COVID-19-related anxiety. Our findings suggest gender-specific interventions for reducing anxiety among older women during the pandemic.

Interestingly, we found that participants residing more than 30 min walking distance to the nearest health centre had a lesser chance of experiencing COVID-19-related anxiety. This can be explained by the fact that people residing close to the health centre are more aware of the deadly effects of COVID-19 as they see people with COVID-19 symptoms visiting health centres and being admitted. Individuals can become anxious about being infected with SARS-CoV-2 virus and experience its harmful effects. Research has also documented that people who had to use health services during the pandemic had serious mental health issues such as fear and anxiety [50, 51].

The present study also revealed that older people’s difficulty in getting medicine during COVID-19 pandemic was significantly associated with COVID-19-related anxiety. We did not find any study to compare this finding with. COVID-19 has resulted in limited access to health services, specifically for chronic diseases in many countries, including Bangladesh [52]. During this pandemic, older adults are expected and encouraged to avoid any places such as clinics, medical centres or pharmacies where they could contract the virus, which coupled with the shortage of medicine supply during the pandemic, resulting in their low access to necessary medicines [53, 54]. Research also documented that COVID-19-related lockdowns and restrictions measures have restricted Bangladeshi older people’s access to routine medical care, thus deteriorating their existing chronic conditions [55,56]. This situation has made them tense, increased their anxiety level and exacerbated psychological health. Our findings suggest focusing on interventions to improve older people’ psychological health during the pandemic.

In our study, the feeling of isolation from others was also significantly associated with a higher level of COVID-19-related anxiety among the participants. Outside of the family, older adults usually depend on peers of a similar age group to spend leisure time and build relationships. The Government of Bangladesh has mostly taken restrictive measures to curb the spread of the SARS-CoV-2 virus [7]. The imposed social isolation and social distancing measures restricted the interaction with peer groups, which is likely to have a large effect on the mental well-being of the older population during the pandemic [41] and affect the psychological well-being of people, especially on the older population [22, 57]. Evidence indicates that anxiety is more likely to occur and worsen in the absence of interpersonal communication [58, 59] and adequate education and training about dealing with COVID-19-related anxiety [60]. Therefore, we recommend providing psychosocial counselling to reduce anxiety among older people who feel isolated during the pandemic.

Our analyses also indicated that participants who felt that they required additional care during the pandemic had a significantly higher likelihood of having COVID-19-related anxiety. Evidence suggests that older persons require various kinds of support, including medical support and financial assistance, because of their diminished functional ability and physical and life-course changes occurring at older ages [61]. During this overwhelming pandemic, it might not always be possible for adult family members to provide support for older adults with increased needs. Such negligence and reality is commonplace in Bangladesh now [62]. As noted above, the advent of COVID-19 pandemic might have aggravated the situation with increased anxiety. Not having the necessary care and support during the crisis period could be more likely to worsen their anxiety level. Our findings highlight the necessity for providing older people with additional care and support during the COVID-19 pandemic.

### Strength and limitations of the study

To the best of our knowledge, this is the first study exploring anxiety related to COVID-19 among the older population in Bangladesh. However, it has several limitations. First, we had to collect information through telephone interviews during the pandemic. Therefore, the sample may not be representative of the entire older population of Bangladesh. Also, there is potential for selection bias as the sampling frame for the study (the pre-existing registry) was prepared with the available household-level information. Secondly, the survey was cross-sectional in nature; therefore, causality cannot be inferred. Thirdly, we could not meet the sample size we calculated, reducing the power due to a low response rate (95%). Finally, we did not qualitatively explore the reasons for the COVID-19-related anxiety among the participants in our study. A mixed-method approach would result in a more in-depth understanding of the issue.

## Conclusion

The findings of the present study have significant policy implications for reducing COVID-19-related anxiety among the older population both during and after the COVID-19 pandemic in Bangladesh. Policy and public health practitioners should consider disseminating validated information related to COVID-19 to avert any possible misconceptions among the people. The study findings highlight the need of increasing awareness about mental health issues during this pandemic among the community and the family members so that older people are given enough importance to receive adequate treatment and support along with community based mental and emotional support. Moreover, in collaboration with other local and international development partners, the Government of Bangladesh should make a concrete strategy to provide mental health support package for the vulnerable segments of the community, including older adults, during the pandemic and beyond. Involving community health workers in targeted interventions can be crucial in providing cost-effective psychosocial support for older people responding to this pandemic [63].

## Supplementary Information

**Additional file 1.** **Annex 1.** Prevalence of anxiety (*N*=1045).

**Additional file 2.** **Annex 2.** Multicollinearity diagnosis results.

## Data Availability

The datasets generated and/or analyzed during the current study are not publicly available due to the organizational policy of the institution undertaking the research but are available from the corresponding author on reasonable request.
