# Supplementary material for: COVID-19 related anxiety and its associated factors: a cross-sectional study on older adults in Bangladesh
Source: BMC Psychiatry. 2022 Nov 28;22:737. doi: 10.1186/s12888-022-04403-2 (PMC9702614; doi:10.1186/s12888-022-04403-2)
Supplement: Supplementary file 1 — Additional file 1. Annex 1. Prevalence of anxiety (N=1045). [file 12888_2022_4403_MOESM1_ESM.docx]

Annex 1: Prevalence of anxiety (N=1045)

| **Characteristics** | **n** | **%** |
| --- | --- | --- |
| Feeling of dizziness, lightheaded, or faint when I read or listened to news about the coronavirus. | 158 | 15.1 |
| Trouble in falling or staying asleep because of thinking about the coronavirus. | 143 | 13.7 |
| Feeling of paralyzed or frozen when thought about or was exposed to information about the coronavirus. | 141 | 13.5 |
| Losing interest in eating when thought about or was exposed to information about the coronavirus. | 139 | 13.3 |
| Feeling nauseous or had stomach problems when thought about or was exposed to information about the coronavirus. | 88 | 8.4 |
