# Supplementary material for: COVID-19 related anxiety and its associated factors: a cross-sectional study on older adults in Bangladesh
Source: BMC Psychiatry. 2022 Nov 28;22:737. doi: 10.1186/s12888-022-04403-2 (PMC9702614; doi:10.1186/s12888-022-04403-2)
Supplement: Supplementary file 2 — Additional file 2. Annex 2. Multicollinearity diagnosis results. [file 12888_2022_4403_MOESM2_ESM.docx]

# Annex 2. Multicollinearity diagnosis results

| **Variable** | **VIF** | **1/V IF** |
| --- | --- | --- |
| Sex | 2.11 | 0.473765 |
| Marital status | 1.64 | 0.607985 |
| Formal schooling | 2.03 | 0.491949 |
| Family size | 3.03 | 0.329795 |
| Walking distance to the nearest health centre | 1.83 | 0.545597 |
| Feeling concerned about COVID-19 | 3.17 | 0.315669 |
| Difficulty in getting medicine during COVID-19 | 1.41 | 0.706769 |
| Feeling isolated from others | 1.57 | 0.637250 |
| Feeling that they required additional care during the pandemic | 1.40 | 0.712798 |
